# Supplementary material for: Relationship between anxiety and internet searches before percutaneous ultrasound-guided diagnostic procedures: A prospective cohort study
Source: PLoS One. 2022 Oct 4;17(10):e0275200. doi: 10.1371/journal.pone.0275200 (PMC9531823; doi:10.1371/journal.pone.0275200)
Supplement: S1 Appendix — (DOCX) [file pone.0275200.s001.docx]

**Name:_________________________________________________________________**

**Age:** ____________

**Gender:** ( ) Male ( ) Female

**- Educational Status:**

( ) Elementary School ( ) Complete ( ) Incomplete

( ) High School ( ) Complete ( ) Incomplete

( ) University ( ) Complete ( ) Incomplete

**- This procedure is funded by the:** ( ) Public Health System ( ) Particular

**- Do you consider that you have regular medical follow-up?** ( ) Yes ( ) No

**- Did you do this procedure today before?** ( ) Yes ( ) No

**- Do you have treatment or follow-up for any type of cancer?**

( ) Yes ( ) No

**- How do you consider yourself health?** ( ) Good Health ( ) Poor Health

**- Do you consider that the physician requesting this procedure:**

( ) provided me with all the necessary information, I have no doubt as to the procedure I will do now.

( ) provided me almost all the necessary information but still have some doubts about the procedure

( ) I have little information about the procedure and I still have many doubts.

**- Choose the phrase that best defines what was passed on to you by the doctor, about the purpose of this exam today:**

( ) to set a treatment for my problem

( ) to find the cause of my problem

( ) Routine examination

( ) do not know the purpose of this examination

**1**

**- Did your doctor pass on any written information about this procedure?**

( ) Yes ( )No

**- A proactive person is one who seeks to anticipate problems, to know situations before they happen...**

**Do you consider yourself proactive in seeking information about your health?**

( ) Yes ( ) No

**- Did you search the internet about your illness?** ( ) Yes ( ) No

**- Have you sought additional information about the procedure on the Internet or requested that any acquaintance/relative search the internet regarding the procedure you will do now?**

( )Yes ( )No

**If your answer is "no" to the question above, please go to the questions on page 3**

**- Why did you search for health information on the Internet?** **(you can check more than one option)**

( ) To take an active role in my treatment, not to confront my doctor, but to ask informed questions and understand what will tell me

( ) To check the competence of my doctor

( ) To seek general information

( ) To find a good doctor

( ) To know what was happening to me, self-diagnose me

( ) To meet people who were going through the same situation as my

( ) To search for different treatment options

( ) None of the above

( ) Another reason:____________________________________________

**- Did you mention in the consultation with the doctor who requested this exam, that you would search the internet for additional information?** ( ) Yes ( ) No

**- In case you didn't mention it, why didn't you?** **(you can check more than one option)**

( ) I found the information of the internet problematic, I did not trust,so I did not speak anything with the doctor

( ) I think it is not the patient's role to talk anything about the disease or treatment with the doctor

( ) I consider that the information I searched on the internet was just generalist, more to understand what my doctor was talking about

( ) The doctor might not like it, did not want to look like he was confronting

( ) My doctor is great, sure knows everything I read and found that it was not necessary to comment

( ) None of the above

**2**

**THE QUESTIONS ARE NOW AS MUCH FOR THOSE WHO RESEARCHED AND FOR THOSE WHO HAVE NOT RESEARCHED THE INTERNET ABOUT THE PROCEDURE YOU WILL DO**

**- Regarding the quality of the information that is on the internet, you consider it:**

( ) Good quality ( ) Medium quality ( ) Poor quality

**- How reliable do you consider the information that is on the internet?**

( ) Very reliable ( ) Reliable ( ) Unreliable ( ) Very unreliable

**- Do you consider that finding reliable information on the Internet is:**

( ) Very easy ( ) Easy ( ) Difficult ( ) Very difficult

**- If you have searched the internet for information about the procedure, and this information has left you:**

**(if you have not searched for information on the internet, SKIP THIS ISSUE)**

( ) Much more confident to do the procedure

( ) More confident to do the procedure

( ) It hasn't changed my confidence to do the procedure

( ) Less confident to do the procedure

( ) Much less confident to do the procedure

**- About the result of this exam you will do:**

Do you think the results of this exam will come out in about how long?

( ) Soon after finishing the procedure, I will wait in the hospital and still today I take the exam home

( ) In less than 2 weeks

( ) In more than 2 weeks

**- Who do you think will analyze your scan under the microscope?**

( ) My doctor, the one who requested the examination

( ) A radiologist, who I will meet when doing the procedure

( ) A doctor I don't know I do not know, a pathologist

( ) A laboratory technician

**3**

**- Why didn't you search the internet for information about the procedure?**

**If you have searched, please skip this question.**

( ) I did not consider the research necessary, because I trust the indication of my doctor;

( ) I didn’t have time, but I would have liked to have done an internet search;

( ) I do not trust the information on the Internet.

( ) I am afraid/afraid of the information I might find on the internet.

**- How do you consider the need for the procedure to which you will undergo?**

( ) Much needed, I'm sure it was correctly indicated for me;

( ) Necessary, although i still have some doubts about the indication of the procedure;

( ) Little needed.

**- You would say that internet research, before some invasive imaging procedure proposed by a doctor, is:**

( ) Indispensable, everyone should consult it before accepting an invasive procedure;

( ) Important but expendable, the doctor's indication should prevail over any information found on the Internet, as it is not always reliable;

( ) totally expendable, searching the internet would only confuse me and general unnecessary questions.

**4**

**(It's almost over, there's only one more page left! Thank you very much)**

**🡪🡪🡪🡪🡪🡪🡪🡪🡪🡪🡪🡪🡪🡪🡪🡪🡪🡪🡪🡪🡪🡪🡪🡪🡪🡪🡪🡪🡪🡪🡪**

**How would you rate the pain you felt during the procedure?**

**( ) 0 I felt no pain**

**( ) 1**

**( ) 2**

**( ) 3**

**( ) 4**

**( ) 5 I felt moderate pain**

**( ) 6**

**( ) 7**

**( ) 8**

**( ) 9**

**( ) 10 It was the worst pain of my life**

**Regarding your expectation of what the procedure would look like, do you think it was:**

**( )** Better than I expected

**( )** Just like I expected

**( )** Worse than I expected

Would you like to make any additional comments?

__________________________________________________________________________________________________________________________________________

**Thank you very much.**

**5**
