# Supplementary material for: Relationship between anxiety and internet searches before percutaneous ultrasound-guided diagnostic procedures: A prospective cohort study
Source: PLoS One. 2022 Oct 4;17(10):e0275200. doi: 10.1371/journal.pone.0275200 (PMC9531823; doi:10.1371/journal.pone.0275200)
Supplement: Attachment — Submitted filename: Response to Reviewers.docx [file pone.0275200.s003.docx]

Additional Editor Comments (if provided):

The article is well written but needs improvement. The reviewers did an excellent job and pointed out the various methodological gaps. Our goal is to give a high quality product to the press.

Authors should make some changes to improve the quality of the data presented. In particular, they must revise the introduction by inserting one or more sentences to bring the average reader closer to the issue. Moreover, they should offer a more articulate discussion of the results and perform a thorough revision of the language. Finally, the text must be revised according to the reviewers' suggestions (it is mandatory). The references suggested by one of the reviewers are excessive. Given the comments I got from the reviewers, my rating is major revision.

**Reviewer #1**

1. The manuscript needs language, grammar, and syntactic editing. The English language usage should be checked by a fluent English speaker (Writing quality is inferior. Numerous grammatical mistakes and meaningless sentences)

- The authors agree with the statements. The entire article was submitted for review by a native English speaker.

2. The primary defect of this study is the debate or argument is not clearly stated in the introduction session. Hence, the contribution is weak in this manuscript. I would suggest the author enhance your theoretical discussion and your debate or argument. The research gap is not evident and appropriate.

- The authors agree. To clarify the central theme of the study, the following excerpt was added to the first paragraph of the introduction: “Although image-guided procedures and the use of the Internet as a resource are ingrained in the modern world, few studies have correlated the two with the presence of anxiety before invasive procedures.”

3. Must add much more explanations and interpretations for the results, which are not enough. It is suggested to compare the results of the present research with some similar studies which is done before (more justification is needed)

- The authors agree. More time was spent on improving the discussion section to bring more clarity when connecting our study with previous publications.

• Should add a flowchart to the article to show the research methodology

- The authors agree and the text was edited to include the flowchart represented in Figure 2.

**Reviewer #2**

This paper is well written and enriches the existing literature. The methodology is sound and has been applied before. The results are presented well and discussed. Therefore, I will recommend the acceptance of the paper.

- The authors are grateful for the comments.

**Reviewer #3:**

I have evaluated this interesting study entitled, "Anxiety and internet research before percutaneous ultrasound-guided diagnostic procedures." is an informative research study. I suggest some minor corrections to check the typo errors in writing to enhance the English quality to reach the scientific merit for the publication of this study.

This article describes that invasive procedures guided by ultrasound (US) are part of the routine medical diagnostic investigation. The lack of knowledge related to technical aspects about them can lead the patient to seek complementary information on the internet, which can trigger anxiety. However, the intersection between the areas of Radiology and Psychology is poorly studied. Here we show the profile of an anxious patient before an US-guided intervention.

I am in favor of this study and will recommend for publication. However, the authors need to revise the manuscript and work according to my suggestions to enhance the quality. I will accept this paper for publication after these minor changes as suggested below.

Introduction and literation sections

I recommend the authors add suggested articles in the introduction and literature sections. These research articles have identified health-related topics I believe it will improve the quality of your work. I strongly suggested them to improve this section a bit more. I advise authors to revisit their introduction and literature sections of the recommended studies and cite these studies to enhance your research study's quality to reach scientific merit for publication.

Wang, C., Wang, D., Duan, K., & Mubeen, R. (2021). Global financial crisis, smart lockdown strategies, and the COVID-19 spillover impacts: A global perspective implications from Southeast Asia. Front Psychiatry, 12, 1-14. doi:10.3389/fpsyt.2021.643783

Abbas, J., Raza, S., Nurunnabi, M., Minai, M. S., & Bano, S. (2019). The Impact of Entrepreneurial Business Networks on Firms’ Performance Through a Mediating Role of Dynamic Capabilities. Sustainability, 11(11), 3006. doi:10.3390/su11113006

NeJhaddadgar, N., Ziapour, A., Zakkipour, G., Abolfathi, M., & Shabani, M. (2020, Nov 13). Effectiveness of telephone-based screening and triage during COVID-19 outbreak in the promoted primary healthcare system: a case study in Ardabil province, Iran. Z Gesundh Wiss, 1-6. <https://doi.org/10.1007/s10389-020-01407-8>

Abbas, J., Aqeel, M., Abbas, J., Shaher, B., A, J., Sundas, J., & Zhang, W. (2019, Feb 1). The moderating role of social support for marital adjustment, depression, anxiety, and stress: Evidence from Pakistani working and nonworking women. J Affect Disord, 244, 231-238. https://doi.org/10.1016/j.jad.2018.07.071

Literature

I want to see publish this creative study after some corrections. I have endorsed this study as; it deserves the merit for publication. However, I suggest the authors make minor corrections according to my advice. Please read the suggested studies and cite them in the introduction, literature, and method sections. How corporate social responsibility, innovation and social media and internet use is helpful. Add few lines in the introduction and literature sections. How companies are practicing CSR, business, entrepreneurial networks with innovation and knowledge sharing to improve the business performance and provide better healthcare medicines?

Azizi, M. R., Atlasi, R., Ziapour, & Naemi, R. (2021). Innovative human resource management strategies during the COVID-19 pandemic: A systematic narrative review approach. Heliyon, 7(6), e07233. doi:10.1016/j.heliyon.2021.e07233

Abbas, J., Zhang, Q., Hussain, I., Akram, S., Afaq, A., & Shad, M. A. (2020). Sustainable Innovation in Small Medium Enterprises: The Impact of Knowledge Management on Organizational Innovation through a Mediation Analysis by Using SEM Approach. Sustainability, 12(6), 2407. doi:https://doi.org/10.3390/su12062407

Azadi, N. A., Ziapour, A., Lebni, J. Y., Irandoost, S. F., & Chaboksavar, F. (2021). The effect of education based on health belief model on promoting preventive behaviors of hypertensive disease in staff of the Iran University of Medical Sciences. Archives of Public Health, 79(1), 69. doi:10.1186/s13690-021-00594-4

Abbas, J., Hussain, I., Hussain, S., Akram, S., Shaheen, I., & Niu, B. (2019). The Impact of Knowledge Sharing and Innovation upon Sustainable Performance in Islamic Banks: A Mediation Analysis through an SEM Approach. Sustainability, 11(15), 4049. doi:10.3390/su11154049

Materials and Methods

The results section of the paper presents a good view of the study. This work presents a notable investigation on a selected topic. I suggest the authors to present high quality graphs. By including some graphical presentations will improve the quality of this study. Please see the proposed studies and see the graphical representation. Improve your work like these studies and cite them in this section.

Paulson, K. R., Kamath, A. M., Alam, T., Bienhoff, K., Abady, G. G., . . . Kassebaum, N. J. (2021). Global, regional, and national progress towards Sustainable Development Goal 3.2 for neonatal and child health: all-cause and cause-specific mortality findings from the Global Burden of Disease Study 2019. The Lancet, 1-36. doi:10.1016/s0140-6736(21)01207-1

Abbas, J., Aqeel, M., Ling, J., Ziapour, A., Raza, M. A., & Rehna, T. (2020). Exploring the relationship between intimate partner abuses, resilience, psychological, and physical health problems in Pakistani married couples: a perspective from the collectivistic culture. Sexual and Relationship Therapy, 35, 1-30. https://doi.org/10.1080/14681994.2020.1851673

Abbas, J., Aman, J., Nurunnabi, M., & Bano, S. (2019). The Impact of Social Media on Learning Behavior for Sustainable Education: Evidence of Students from Selected Universities in Pakistan. Sustainability, 11(6). https://doi.org/10.3390/su11061683

Abbas, J., Aqeel, M., Jaffar, A., Nurunnabi, M., & Bano, S. (2019, 2019/07/01). Tinnitus perception mediates the relationship between physiological and psychological problems among patients. Journal of Experimental Psychopathology, 10(3), 2043808719858559. <https://doi.org/10.1177/2043808719858559>

- The authors agree and the text was edited to include the flowchart represented in Figure 2.

Discussion

I suggest the authors to discuss the effects of the COVID-19. I suggest you to cite these studies. Read the proposed studies to improve your results and discussion section. See the recommended studies and improve your sections.

Su, Z., McDonnell, D., Wen, J., Kozak, M., Šegalo, S., . . . Xiang, Y.-T. (2021). Mental health consequences of COVID-19 media coverage: the need for effective crisis communication practices. Globalization and Health, 17(1), 4. doi:10.1186/s12992-020-00654-4

Aqeel, M., Shuja, K. H., Rehna, T., Ziapour, A., Yousaf, I., & Karamat, T. (2021). The Influence of Illness Perception, Anxiety and Depression Disorders on Students Mental Health during COVID-19 Outbreak in Pakistan: A Web-Based Cross-Sectional Survey. International Journal of Human Rights in Healthcare, 14, 1-14.

Abbas, J. (2020). The Impact of Coronavirus (SARS-CoV2) Epidemic on Individuals Mental Health: The Protective Measures of Pakistan in Managing and Sustaining Transmissible Disease. Psychiatr Danub, 32(3-4), 472-477. <https://doi.org/10.24869/psyd.2020.472>

- The authors are grateful for the constructive feedback and have read all the above studies. After careful consideration we selected the following articles to include in our study. The first one was entitled “Abbas, J., Aqeel, M., Abbas, J., Shaher, B., A, J., Sundas, J., & Zhang, W. (2019, Feb 1). The moderating role of social support for marital adjustment, depression, anxiety, and stress: Evidence from Pakistani working and nonworking women. J Affect Disord, 244, 231-238. https://doi.org/10.1016/j.jad.2018.07.071”. It fits perfectly with our argument that correlates higher anxiety in the female gender, as it follows: “Preoperatively, multiple parameters were evaluated including: patients’ age, sex gender, occupation, education level, type of surgery, and whether they patient had previously undergone a similar procedure. Respondents completed the STAI questionnaire for anxiety psychometry in the waiting room right before their intervention. Of all the parameters examined, only the female gender was found to be positively correlated with anxiety levels. This finding has also been reported in other parts of the world [10]. Jafar et al. used STAI to evaluate 300 pre-surgical patients in Pakistan and found higher levels of anxiety in females. However, considering anxiety questionnaires are self-administered, female patients may be more inclined to admit to having anxiety than males patients. It is therefore necessary to exercise caution before stating that these data represent an innate difference in anxiety levels between genders [11].”

Conclusion

I suggest you make a separate heading of the conclusion and do not mix it with implications.

Policy Recommendations

I again recommend you to make a separate heading of the Policy Recommendations.

- Although the author agrees with the comment and think it would be more coherent to separate the policy recommendations and implications from the conclusion, this paper follows the recommendations and structure set out in PLOSONE guideline.

The conclusion section is acceptable. Overall, this presents a good piece of research work. I recommend that authors do a little more work and revise this article accordingly. I suggest the authors check English quality and fix some weak sentences. If you have already taken English editing service, ask them to recheck the quality to meet scientific merit for publication. I endorse this manuscript for publication after minor corrections, as suggested.

- The authors agree. To clarify the central theme of the study, the following excerpt was added to the first paragraph of the introduction: “Although image-guided procedures and the use of the Internet as a resource are ingrained in the modern world, few studies have correlated the two with the presence of anxiety before invasive procedures.”. Additionally, the entire article was submitted for review by a native English speaker.

**Reviewer #4**

Thank you for submitting the manuscript. I have read your manuscript very carefully. The theme you are addressing is an important one that investigates the doctor-patient relationship. I am convinced that small adjustments can increase the quality and readability of the paper.

1. First of all I would like you to enter the protocol number of the approval by the Ethics Committee of the study.

- The authors agree and added the following text: “(certificate number approval by the ethics committee 66022517.8.0000.5432, appraisal 2.063.731).” - Study Design.

2. I would also like to have more information about the patient's medical history. I'll explain. The paper does not talk about the psychological history of patients, but only about their self-definition as pro-active or not. This definition is too reductive to understand the psychological situation of the patients involved. The psychological history of the subjects would provide us with important information to better understand the possible origin of anxiety and possible confounding factors. Have any traumatic factors been investigated in the history of the subjects (war veterans, child abuse, road accidents ...)? Have you asked about the possible use of anxiolytic drugs? Have you asked if the subjects have ever been in psychotherapy? If you have this information, in my opinion, it must be explained in the paper and it must be correlated with the results. If, on the other hand, you do not have one, it is necessary to insert this gap among the limitations of the study. I am sure that with these small suggestions the paper can be improved. I hope these comments are helpful to you.

- This observation is very important and the authors are grateful. During the study, we were careful to look for appointments with psychologists or psychiatrists by the participants. Despite not being an inclusion/exclusion criterion, we found that no patient had had an appointment with these professionals at the hospital where the invasive procedure was performed. This does not prevent an important limitation of our study since aspects of psychiatric illnesses were not directly addressed or questioned. These points have been added to the Discussion session, in limitations, as follows: “Finally, there were no direct questions included regarding the presence of psychiatric illness among study participants. While the authors found no evidence of consultations with clinical psychologists or psychiatrists in the patients’ electronic medical records, participants were not directly asked about symptoms or previous traumatic events.”
