# Supplementary material for: Relationship between anxiety and internet searches before percutaneous ultrasound-guided diagnostic procedures: A prospective cohort study
Source: PLoS One. 2022 Oct 4;17(10):e0275200. doi: 10.1371/journal.pone.0275200 (PMC9531823; doi:10.1371/journal.pone.0275200)
Supplement: Attachment — Submitted filename: Response to Reviewers.docx [file pone.0275200.s004.docx]

**Editor Comments**

I suggest further changes

**1.** TITLE. Include the type of the study and setting.. For example: "A prospective cohort study ..."

- The study title has been changed to: “Relationship Between Anxiety and Internet Searches Before Percutaneous Ultrasound-guided Diagnostic Procedures: A Prospective Cohort Study”

**2.** ABSTRACT. Include p-values

- P-values were included.

**3.** LIMITATIONS (It deserves a dedicated paragraph). Emphasizes that cancer patients are individuals with a significant anxiety burden.

- Prior to specific limitations session, the following was added: “It should also be noted that there is a high prevalence of psychiatric disorders such as depression and anxiety amongst cancer patients [22]. This universe requires understanding from health professionals involved in care, despite the large knowledge gap in the medical literature and the absence of specific guidelines on the subject [23]. While our study advances understanding of image-guided diagnostic procedures, it is important to note cancer patients are individuals with a significant anxiety burden that requires further investigation~~s~~ on the subject.

**3.** CONCLUSION. Add "Despite limitations, this study shows that ..."

- Added phrase as follows: “Despite limitations, this study shows that higher anxiety scores before invasive US-guided procedures were found in female patients, in those who reported having had received insufficient information from their referring physician, and in those who considered online information to be unreliable or difficult to access. The recognition of this profile can guide measures to reduce anxiety in patients who will undergo an US-guided invasive procedure, by improving patient-physician communication and providing accurate and easily accessible information online.”

**4.** REFERENCES. Many references are more than 20 years old. This is a gap for the article. Add, other more recent references:

a. about anxiety: doi: 10.1097/RMR.0000000000000238; doi 10.18632/oncotarget.17238; doi: 10.1002/da.23115 b. about internet-based patients' searching: doi: 10.1176/appi.ps.201800495; doi: 10.1371/journal.pone.0261471.

- The authors agree with this observation. The reference “Bull Med Libr Assoc. **2001** Oct;89(4):397-9. PMID: 11837263; PMCID: PMC57970” has been replaced by “Top Magn Reson Imaging. **2020** Aug;29(4):197-201. doi: 10.1097/RMR.0000000000000238. PMID: 32472820”. Another updated reference was added (Psychiatr Serv. **2019** Apr 1;70(4):324-328.”).
- We are especially grateful for the suggested article " PLoS One. **2021** Dec 31;16(12):e0261471" as it fits properly into the following manuscript excerpt: “Three potential responses to patients who utilize online searches prior to consultation were discussed: 1) the healthcare professional feels threatened by the information and responds by defensively stating his "expert opinion"; 2) the healthcare professional and the patient cooperate in the analysis of online information; 3) the healthcare professional guides patients to reliable sites on the Internet. Previous studies suggest positive results when the information brought from the Internet by the patient is discussed together with the doctor [21]. On the other hand, it is precisely in this third response that our study supports and leads us to formulate a question yet to be answered: can proactivity be encouraged during consultations? If so, could it result in reduced anxiety before US-guided procedures?”

**Finally**, I suggest another round of lexical correction. The style still doesn't sound.

- A new spelling and writing style review was requested from a professional who is native and fluent in the English language.
