# Supplementary material for: Relationship between anxiety and internet searches before percutaneous ultrasound-guided diagnostic procedures: A prospective cohort study
Source: PLoS One. 2022 Oct 4;17(10):e0275200. doi: 10.1371/journal.pone.0275200 (PMC9531823; doi:10.1371/journal.pone.0275200)
Supplement: Attachment — Submitted filename: Response to Reviewers.docx [file pone.0275200.s005.docx]

**Reviewer #5**

* It is a very interesting and very current topic. Health systems must find strategies to increase the well-being of their patients. This work can help to find these strategies, after identifying the causes.

**1.** *Despite not being native to the English language, it seems to me that English should be revised.

- The authors agree with the statements. The entire article was submitted for review by a native English speaker.

**2.** *On page 12, section "Statistical Methods", authors should add, right after the phrase "The non-parametric Mann-Whitney test was used to compare two subgroups, and the Kruskal-Wallis test was used when three subgroups were analyzed.", the following:

"In the 3 group comparison, whenever statistically significant differences were detected, the Kruskal-Wallis multiple comparison test was used."

Authors should not use the Mann-Whitney test to compare groups 2 to 2, as this increases the probability of type I error from 0.05 to 0.143 (as it is related to the number of comparisons to be performed). They should use the Kruskal-Wallis multiple comparison tests, already available in SPSS version 20.0.

- The authors agree with the statements. The suggested sentence was included in its entirety in the manuscript.

**3.** * The titles of tables 2, 3, 4 and 5 are not correct. In any of the tables, what the authors are doing is comparing anxiety levels (STAI-State and STAI-Trait) between groups. They are not studying the correlation.

- The authors agree with the indicated inadequacy. The term *relationship* has been changed to *correlation*.

**Reviewer #6**

It seems, the manuscript do not follow scientific writing for an article such as topic presentations and study design and figure 1, raw presentation.

- The authors appreciate the reviewer's comment. However, we did our best to follow scientific methodology, especially in writing. After going through corrections from 7 different reviewers and 2 editors, we believe we have done a good job. Thanks again for the comment.

**Reviewer #7**

It is a current exciting topic and manuscript. While not the most original work, it adds exciting data concerning patient-centered care during diagnostic procedures. It provides essential information to oncology specialty professionals and general practice. However, the manuscript presents some relevant weaknesses.

**1.** A linguistic revision is suggested to make the reading more accessible and precise.

- The authors agree with the statements. The entire article was submitted for review by a native English speaker.

**2.** Abstract: Reduce background (the last two sentences are not needed) and improve methods, results, and conclusion

- The authors are grateful for the observations made by the reviewer. The abstract was reduced, and the other sections were revised to clarify the ideas presented.

**3.** Introduction: The introduction should be improved with more references related to diagnostic medical procedures and online search anxiety. There are scientific papers available.

- The authors agree with the observed fact and chose to add the following article to the manuscript: "Swire-Thompson B, Lazer D. Public Health and Online Misinformation: Challenges and Recommendations. Annu Rev Public Health. 2020 Apr 2;41:433-451 doi: 10.1146/annurev-publhealth-040119-094127. Epub 2019 Dec 24. PMID: 31874069". It is an excellent source that supports the misinterpretations that online information can lead to.

**4.** Methods: Please organize better methods section regarding Sample or participants, measures (information regarding STAI is repetitive), proceedings (including dates for data collecting), ethical considerations, and Statistical analyses.

- The authors eliminated some repetitive information about the STAI questionnaire and also added the study data collection date to the manuscript in the Methods / Study Design section.

**5.** Results: Did the authors considered performed Hierarchical linear regression analyses to explore potential predictors of patients’ anxiety before US-guided invasive procedures?

Considering STAI_T, authors could also use descriptive data of patients with clinically relevant anxiety and without clinically relevant anxiety.

- The authors are grateful for the questions raised. Hierarchical linear regression analysis was considered, however, as it is a topic such as anxiety, with countless variables, the authors opted for a statistical method rather than a model comparison. The STAI_T was used in our study basically because it was attached to the STAI_S in its original questionnaire. However, as the image-guided invasive procedure is a one-off event, its use could have been dispensed with. There were no significant correlations with this questionnaire. We chose to keep it in the manuscript precisely to raise this discussion.

**6.** Discussion: The discussion tries to be exhaustive, with references to other studies. However, the complete information related to cancer is not clear since the current sample did not have a cancer diagnosis. This need to be clarified perhaps in the methods section

making it very long and with some unnecessary indications (for example, values of the statistics of the studies with which it compares its results). The discussion should also include limitations and indications for future studies.

- The authors are grateful for the comments raised by the reviewer. The sample of participants included cancer patients (45 patients, about 34% of the total), as shown in Table 1. There was no correlation with anxiety levels , as shown in the Results section: "There were no significant associations between STAI anxiety scores and age, education level, age, past experiences of health treatment of the medical status, or proposed procedure or subjective assessments of the regularity of care (P > 005)." As the reviewer correctly pointed out, this issue needs clarification, this point being addressed in the Discussion, in limitations, in the following excerpt: "It should also be noted that there is a high prevalence of psychiatric disorders such as depression and anxiety amongst cancer patients [23 ]. Despite the large knowledge gap in the medical literature and the absence of specific guidelines on the subject [24], health professionals in care are required to understand how to approach said conditions."

A linguistic revision is suggested to make the reading more accessible and precise.

- The authors agree with the statements. The entire article was submitted for review by a native English speaker.
