# Supplementary material for: Relationship between anxiety and internet searches before percutaneous ultrasound-guided diagnostic procedures: A prospective cohort study
Source: PLoS One. 2022 Oct 4;17(10):e0275200. doi: 10.1371/journal.pone.0275200 (PMC9531823; doi:10.1371/journal.pone.0275200)
Supplement: Attachment — Submitted filename: Response to Reviewers.docx [file pone.0275200.s006.docx]

**Reviewer #5**

* The article has improved substantially. However, there are still some details that must be corrected, namely:

**1.** * Whenever authors refer to the p-value, it must be in lower case, p instead of P. Authors should review the entire text.

- The authors are grateful for the observation made. The entire manuscript was revised and the p-values corrected.

**2.** * The titles of tables 2, 3, 4 and 5 are not correct, as they are not evaluating the form and intensity of the relationship between two variables, but the comparison of the values of a variable (which is quantitative) between the categories of a qualitative variable. Therefore, instead of correlation, there should be a comparison, for example comparison of the state of anxiety (assessed by STAI) between ...

- The authors agree with the observations made. Conceptual changes were made to the table titles.

**Reviewer #6**

**1.** It seems, the manuscript needs to rewrite or revise by an scientific writer for scientific article in order to be condense, clear methodology even the name of method " One hundred and thirty-three patients were evaluated prospectively". Repeatable of the research work is important. Sampling and research community, response rate are important as well.

- The authors are again grateful for the reviewer's notes. The entire article was reviewed and the necessary changes were made to clarify the scientific language.
